# Adaptive Pacing, Cognitive Behaviour Therapy, Graded Exercise, and Specialist Medical Care for Chronic Fatigue Syndrome: A Cost-Effectiveness Analysis

**DOI:** 10.1371/journal.pone.0040808

**Published:** 2012-08-01

**Authors:** Paul McCrone, Michael Sharpe, Trudie Chalder, Martin Knapp, Anthony L. Johnson, Kimberley A. Goldsmith, Peter D. White

**Affiliations:** 1 Centre for the Economics of Mental and Physical Health, Health Service and Population Research Department, Institute of Psychiatry, King’s College London, London, United Kingdom; 2 University Department of Psychiatry, University of Oxford, Oxford, United Kingdom; 3 Academic Department of Psychological Medicine, King’s College London, London, United Kingdom; 4 Personal Social Services Research Unit, London School of Economics, London, United Kingdom; 5 MRC Biostatistics Unit, Institute of Public Health, Cambridge, United Kingdom; 6 MRC Clinical Trials Unit, London, United Kingdom; 7 Biostatistics Department, Institute of Psychiatry, King’s College London, London, United Kingdom; 8 Centre for Psychiatry, Wolfson Institute of Preventive Medicine, Barts and the London School of Medicine and Dentistry, Queen Mary University of London, London, United Kingdom; Erasmus University Rotterdam, The Netherlands

## Abstract

**Background:**

The PACE trial compared the effectiveness of adding adaptive pacing therapy (APT), cognitive behaviour therapy (CBT), or graded exercise therapy (GET), to specialist medical care (SMC) for patients with chronic fatigue syndrome. This paper reports the relative cost-effectiveness of these treatments in terms of quality adjusted life years (QALYs) and improvements in fatigue and physical function.

**Methods:**

Resource use was measured and costs calculated. Healthcare and societal costs (healthcare plus lost production and unpaid informal care) were combined with QALYs gained, and changes in fatigue and disability; incremental cost-effectiveness ratios (ICERs) were computed.

**Results:**

SMC patients had significantly lower healthcare costs than those receiving APT, CBT and GET. If society is willing to value a QALY at £30,000 there is a 62.7% likelihood that CBT is the most cost-effective therapy, a 26.8% likelihood that GET is most cost effective, 2.6% that APT is most cost-effective and 7.9% that SMC alone is most cost-effective. Compared to SMC alone, the incremental healthcare cost per QALY was £18,374 for CBT, £23,615 for GET and £55,235 for APT. From a societal perspective CBT has a 59.5% likelihood of being the most cost-effective, GET 34.8%, APT 0.2% and SMC alone 5.5%. CBT and GET dominated SMC, while APT had a cost per QALY of £127,047. ICERs using reductions in fatigue and disability as outcomes largely mirrored these findings.

**Conclusions:**

Comparing the four treatments using a health care perspective, CBT had the greatest probability of being the most cost-effective followed by GET. APT had a lower probability of being the most cost-effective option than SMC alone. The relative cost-effectiveness was even greater from a societal perspective as additional cost savings due to reduced need for informal care were likely.

## Introduction

Chronic fatigue syndrome (CFS) describes a condition of chronic disabling fatigue for which there is no other explanatory condition. It has a prevalence of 0.2–2.6% of people worldwide [1]. CFS can disrupt employment and necessitates support from families in addition to formal healthcare [2], [3]. Therapies have been developed for CFS, particularly cognitive behaviour therapy and graded exercise therapy [4]. In the PACE trial we compared the clinical effectiveness of these two therapies with adaptive pacing therapy, when added to specialist medical care, and with specialist medical care alone [5]. Adding cognitive behaviour therapy or graded exercise therapy to specialist medical care was found to be more effective in reducing both fatigue and disability than adding adaptive pacing therapy or specialist care alone.

The aims of this paper are to compare (i) the one-year service and societal costs associated with specialist medical care (SMC) plus cognitive behaviour therapy (CBT), SMC plus graded exercise therapy (GET), SMC plus adaptive pacing therapy (APT) and SMC alone, and (ii) the one-year cost-effectiveness of CBT, GET, APT and SMC in terms of gains in quality-adjusted life years (QALYs) and reductions in fatigue and disability.

## Methods

### Study Design and Participants

PACE was a parallel four-arm, multi-centre, randomised controlled trial with participants recruited from consecutive new outpatients attending six secondary care specialist CFS clinics in the UK. Participants were selected using the Oxford diagnostic criteria for CFS which required disabling fatigue to be the primary problem, in the absence of an exclusionary medical or psychiatric diagnosis [6]. Other inclusion criteria were: aged 18 years or more, a binary score of 6 or more out of 11 on the Chalder fatigue questionnaire [7], and a score of 65 or less out of 100 on the Short Form-36 physical function sub-scale [8]. Exclusionary criteria and allocation procedures have previously been described [5]. Written consent was obtained from all participants. Ethical approval was given by the West Midlands Multi-centre Research Ethics Committee (MREC 02/7/89).

### Interventions

Treatment manuals are available at www.pacetrial.org. These specified at least three sessions of SMC for all participants and up to 15 individual therapy sessions for those allocated to APT, CBT and GET. SMC was provided by CFS doctors and consisted of provision of information about CFS, advice for coping, and symptomatic pharmacotherapy. APT involved management of energy expenditure and activity, under occupational therapist supervision, and aimed at helping the patients to adapt by ‘pacing’ their activity. CBT, delivered by clinical psychologists or nurse therapists, aimed to change behavioural and cognitive factors assumed to be responsible for perpetuating symptoms and disability. GET, delivered by physiotherapists, aimed to return the participant to an appropriate level of physical activity by increasing exercise in a gradual and personalised manner.

### Outcomes

Quality-adjusted life years (QALYs) were generated from the EQ-5D health-related quality of life questionnaire at baseline, and at 12, 24 and 52 weeks after randomisation [9]. The EQ-5D measures the following domains: mobility, self-care, usual activities, pain/discomfort, anxiety/depression. Each was coded as 1 (no problem), 2 (moderate problems) or 3 (severe problems) and UK-specific utility weights attached [10]. The accrual of QALYs was calculated using area under the curve, assuming a linear change between each available time point. Differences in baseline utility scores were controlled for when making comparisons between treatment groups [11].

Interpretation of condition specific outcome measures in economic evaluations is difficult and to aid interpretability we assessed the cost per person achieving a clinically important change. Two variables were created to indicate whether patients achieved a two-point improvement on the Chalder fatigue questionnaire (CFQ) and an eight-point change on the Short Form-36 physical function sub-scale (SF36 PF) [7], [8]. These changes were assumed to be clinically significant by White et al [5].

### Service Use and Costs

In our analysis we adopted both a healthcare and a societal perspective. (The latter includes lost employment and unpaid informal care as well as health costs.) The number and duration of APT, CBT, GET and SMC treatment sessions actually delivered were recorded and time added for preparation, related correspondence, and supervision. It was assumed that the ratio of time spent on patient-related versus support activities was 1∶0.3 and that capital and administrative overheads were 46% [12]. The cost per hour of therapy was £110 for CBT and £100 for APT and GET. The cost of SMC was based on the cost per hour of consultant physician time in face-to-face contact with patients, which was £169 [12].

Services used during the six months before randomisation and during the 12 months after randomisation were measured with the Client Service Receipt Inventory (CSRI) [13]. (The CSRI was used at 6 and 12 months after randomisation and the data were combined.) Services are listed in Appendix S1. Where available, unit costs were obtained from nationally recognised sources [12], [14]. Specific types of medication (analgesics, antidepressants, anxiolytics, and hypnotics) were recorded and average costs assumed for each type [15].

Unpaid informal care from family/friends was measured by asking patients how many hours of care were provided because of fatigue. Alternative methods exist for valuing informal care, with the opportunity cost and replacement cost approaches being the most recognised. We adopted the former and valued informal care at £14.60 per hour based on national mean earnings [16]. Days lost by patients from work, and reduced hours while at work, due to fatigue were also recorded. The human capital approach was used with the value of lost work-time to society assumed to be reflected by national mean age and gender-specific wage rates and combined with the lost employment data to generate lost production costs [16].

We excluded welfare benefits or payments from other sources such as private pensions and income protection schemes from the economic costs. However, we do report the receipt of these given that they are important financial outlays.

### Analysis

Comparisons were made between SMC alone and each of APT, CBT and GET. Healthcare, informal care and societal costs (including lost production and informal care) during the 12 months after randomisation in each treatment group were compared using regression models controlling for baseline costs and clustering for centre. Confidence intervals were generated around the cost differences using non-parametric bootstrapping [17].

Cost-effectiveness analyses combining data on incremental costs and outcomes can be compared for each treatment group in turn against each of the others. If costs for one treatment were lower and outcomes better than another treatment, that treatment was defined as ‘dominant’. If costs were higher (lower) and outcomes better (worse) then an incremental cost-effectiveness ratio (ICER) was calculated (i.e. extra cost divided by extra outcome). The ICER indicates the cost per QALY gained or cost per unit reduction in fatigue or disability for one treatment against another. The threshold used to assess the QALY ICERs was £30,000. ICERs constructed with the CFQ and SF-36 PF data used the differences in proportions achieving clinically important changes. The resultant ICER indicates the cost of one extra person achieving such a change as a result of using APT, CBT or GET in addition to SMC compared to SMC alone.

Interpretation of the cost-effectiveness results was made using cost-effectiveness acceptability curves [18]. Net benefit values were computed for each study participant, defined as the value of a QALY multiplied by the number of QALYs gained minus the cost (from both healthcare and societal perspectives). We used QALY values ranging from £0 to £60,000 in increments of £5000. For each QALY value, regression models were used to determine the difference in net benefit between the four treatment arms, controlling for baseline utility and costs. Bootstrapping with 1000 resamples allowed the proportion of resamples showing APT, CBT, GET and SMC as having the highest net benefit (and to be most cost-effective) to be computed and plotted.

Sensitivity analyses were conducted around key parameters in the analyses about which assumptions had been made. Specifically we (i) estimated the cost of therapy required to reverse the findings from the initial analysis, (ii) used the minimum wage rate (£5.93 per hour) and the unit cost of a homecare worker to value informal care, (iii) used the minimum wage rate to value lost production, (iv) reduced the cost of standardised medical care by 50% to reflect the possibility of it being provided by a less senior doctor.

### Role of the Funding Source

The sponsors of the study had no role in study design, data collection, data analysis, data interpretation, or writing of the report. All named authors had access to the data, commented drafts, and approved the final report. Members of the writing group had responsibility for submitting the report, and PM had final responsibility for the decision to submit for publication.

**Table 1:** Baseline demographic and clinical data.

Treatment (n)	APT (159)	CBT (161)	GET (160)	SMC (160)
*Demographic data*				
Age (years): Mean (SD)	39 (11)	39 (12)	39 (12)	37 (11)
Female	121 (76)	129 (80)	123 (77)	122 (76)
Caucasian	146 (92)	151 (94)	148 (93)	150 (94)
Any ME group membership	31 (19)	26 (16)	25 (16)	23 (14)
*Clinical data*				
International CFS criteria	107 (67)	106 (66)	106 (66)	108 (68)
London ME criteria	81 (51)	84 (52)	84 (53)	80 (50)
Any depressive disorder	54 (34)	52 (32)	54 (34)	53 (33)
Any psychiatric disorder	75 (47)	75 (47)	73 (46)	77 (48)
Duration of illness (months) Median (25^th^, 75^th^ quartiles)	33 (16, 69)	36 (16, 104)	35 (18, 67)	25 (15, 57)
BMI (m^2^/Kg) Mean (SD)	25.9 (5.5)	25.4 (5.2)	25.5 (4.6)	25.1 (4.5)

Data are N (%) unless otherwise stated.

APT  =  adaptive pacing therapy, CBT  =  cognitive behaviour therapy, GET  =  graded exercise therapy, SMC  =  specialist medical care alone, ME  =  myalgic encephalomyelitis, BMI  =  body mass index.

## Results

### Sample Characteristics

641 patients were recruited, one of whom withdrew consent. Demographic and clinical characteristics of participants were similar across treatments, apart from a shorter duration of illness in SMC (Table 1). Costs and QALYs were available for 570 (89%) participants (ranging from 85% GET to 93% SMC). Those for whom both cost and QALY data were available were significantly more likely to be of Caucasian ethnicity (94.6% v 86.4%, fishers exact test p = 0.027). There were no other statistically significant differences in the background characteristics shown in Table 1. Further details of the sample have been reported previously [5].

### Service Use and Lost Employment before Randomisation

During the 6 months before randomisation, most participants had used primary care services and over 40% other (secondary care) doctors (Table 2). Around two-thirds had used other health service professionals, between one-quarter and one-third had used complementary healthcare practitioners; most used medication. The intensity of service use (mean contacts for those using them) revealed no substantial differences between treatment groups. Lost employment was common in all treatment groups.

**Table 2:** Service use and lost employment at baseline and follow-up.

	6-month pre-randomisation period							
	N (%) using services				Mean (sd) contacts per user			
Service	APT (n = 159)	CBT (n = 161)	GET (n = 160)	SMC (n = 160)	APT (n = 159)	CBT (n = 161)	GET (n = 160)	SMC (n = 160)
Primary care	154 (97)	154 (96)	157 (98)	156 (98)	5.3 (3.9)	5.6 (4.6)	5.4 (3.3)	5.9 (4.5)
Other doctor	66 (42)	68 (42)	71 (44)	71 (44)	2.3 (2.0)	2.5 (2.0)	2.0 (1.4)	2.3 (2.5)
Health professional	95 (60)	105 (65)	109 (68)	109 (68)	3.7 (5.1)	3.8 (5.5)	3.2 (3.8)	3.6 (5.6)
Inpatient^b^	6 (4)	7 (4)	10 (6)	15 (9)	1.3 (0.5)	1.7 (1.1)	1.0 (0.0)	2.0 (2.3)
Accident and emergency	13 (8)	16 (10)	20 (13)	19 (12)	1.3 (0.9)	1.1 (0.3)	1.3 (0.9)	1.4 (0.6)
Medication^b^	118 (74)	130 (81)	121 (76)	122 (76)	–	–	–	–
Complementary healthcare	50 (31)	41 (25)	55 (34)	52 (33)	7.1 (6.8)	6.5 (6.3)	8.2 (9.3)	7.5 (8.7)
Other health/social services	157 (99)	159 (99)	160 (100)	160 (100)	16.0 (9.7)	16.8 (9.6)	16.2 (8.0)	16.0 (8.9)
Informal care^d^	118 (74)	106 (66)	120 (75)	128 (80)	11.5 (11.1)	10.4 (8.3)	9.6 (9.3)	12.3 (13.7)
Lost employment^e^	127 (80)	135 (84)	132 (83)	137 (86)	81.0 (53.3)	85.3 (52.7)	83.0 (53.7)	75.5 (50.6)
	**12-month post-randomisation period**							
	**N (%) using services**				**Mean (sd) contacts per user**			
**Service** a	**APT (n = 146)**	**CBT (n = 145)**	**GET (n = 140)**	**SMC (n = 148)**	**APT (n = 159)**	**CBT (n = 161)**	**GET (n = 160)**	**SMC (n = 160)**
Primary care	134 (92)	134 (92)	134 (96)	139 (94)	7.1 (5.7)	6.6 (5.6)	6.3 (3.9)	7.0 (4.5)
Other doctor	60 (41)	71 (49)	65 (46)	67 (45)	2.4 (2.2)	2.5 (2.0)	3.1 (2.9)	3.2 (5.6)
Health professional	109 (75)	110 (76)	115 (82)	118 (80)	5.3 (7.9)	4.4 (5.9)	5.6 (8.3)	4.7 (4.7)
Inpatient^b^	17 (12)	16 (11)	21 (15)	18 (12)	3.2 (3.5)	1.4 (0.7)	2.2 (2.4)	2.2 (2.3)
Accident and emergency	26 (18)	22 (15)	14 (10)	19 (13)	1.1 (0.3)	1.4 (0.7)	1.6 (1.2)	1.8 (1.2)
Medication^c^	112 (77)	117 (81)	108 (77)	124 (84)	–	–	–	–
Complementary healthcare	42 (29)	32 (22)	39 (28)	47 (32)	8.5 (9.6)	10.0 (14.4)	12.3 (12.0)	10.2 (11.1)
Informal care^d^	108 (74)	96 (66)	98 (70)	111 (75)	11.0 (10.7)	8.0 (8.6)	7.7 (8.7)	11.4 (11.6)
Other health/social services	108 (74)	110 (76)	106 (76)	105 (71)	6.3 (6.7)	6.3 (9.2)	7.3 (8.1)	7.6 (10.2)
Therapy	146 (100)	145 (100)	140 (100)	–	13.0 (2.4)	13.3 (2.4)	12.9 (2.5)	–
Standardised medical care	146 (100)	145 (100)	138 (99)	148 (100)	3.6 (1.7)	3.7 (2.2)	3.6 (1.4)	5.0 (2.7)
Informal care^d^	108 (74)	96 (66)	98 (70)	111 (75)	11.0 (10.7)	8.0 (8.6)	7.7 (8.7)	11.4 (11.6)
Lost employment^e^	124 (86)	122 (84)	118 (86)	130 (89)	148.6 (109.2)	151.0 (108.2)	144.5 (109.4)	141.7 (107.5)

a see Appendix S1 for services included in these categories,

b contacts measured in bed days,

c quantity unreported as average cost assumed for each patient using medication,

d contacts measured in weekly hours,

e days lost from work.

APT  =  adaptive pacing therapy, CBT  =  cognitive behaviour therapy, GET  =  graded exercise therapy, SMC  =  specialist medical care alone.

### Service Use and Lost Employment after Randomisation

During the 12 months after randomisation, SMC participants had a higher mean number of specialist medical care contacts than those allocated to additional therapy. The number of therapy contacts did not differ between APT, CBT and GET groups. Other service use did not greatly differ between treatments during this period, although informal care hours for APT and SMC were higher than for CBT and GET. There was no clear difference between treatments in terms of lost employment.

### Costs

Costs are shown in Table 3. Controlling for baseline, healthcare costs after randomisation were significantly lower for SMC than for APT (difference £840, 95% CI £637 to £1117), CBT (difference £904, 95% CI £613 to £1205) and GET (difference £829, 95% CI £534 to £1165). The differences between the APT, CBT and GET groups were small and non-significant.

**Table 3:** Service costs at baseline and follow-up.

	6-month pre-randomisation period			
	Mean (SD) cost (2009/10 UK pounds)			
Service^a^	APT (n = 159)	CBT (n = 161)	GET (n = 160)	SMC (n = 160)
Primary care	163 (177)	150 (166)	148 (146)	171 (160)
Other doctor	159 (339)	200 (551)	137 (232)	145 (338)
Health professional	85 (193)	121 (297)	98 (213)	86 (163)
Inpatient	13 (72)	23 (129)	22 (90)	69 (352)
Accident and emergency	10 (42)	10 (32)	16 (52)	16 (47)
Medication	46 (38)	48 (37)	45 (36)	41 (33)
Complementary healthcare	89 (201)	67 (169)	112 (266)	98 (242)
Other health/social services	187 (649)	136 (185)	131 (137)	145 (201)
Informal care	3233 (4097)	2601 (3181)	2719 (3441)	3732 (5018)
Lost employment	7822 (6770)	7978 (6282)	8095 (6745)	7499 (6094)
*Total health costs*	*752 (901)*	*755 (857)*	*709 (485)*	*770 (736)*
*Total societal costs*	*11,807 (8223)*	*11,333 (7452)*	*11,523 (7705)*	*12,001 (8510)*
	**12-month post-randomisation period**			
	**Mean (SD) cost (2009/10 UK pounds)**			
**Service** a	**APT (n = 146)**	**CBT (n = 145)**	**GET (n = 140)**	**SMC (n = 148)**
Primary care	178 (217)	165 (161)	170 (165)	198 (186)
Other doctor	177 (416)	169 (332)	188 (319)	238 (877)
Health professional	120 (164)	123 (178)	152 (230)	168 (345)
Inpatient	142 (619)	54 (180)	132 (500)	99 (414)
Accident and emergency	19 (44)	20 (53)	15 (57)	22 (71)
Medication	70 (68)	78 (68)	70 (54)	77 (65)
Complementary healthcare	98 (256)	89 (315)	137 (335)	129 (313)
Other health/social services	141 (281)	111 (299)	146 (242)	118 (251)
Therapy^b^	1040 (275)	1198 (366)	935 (300)	–
Standardised medical care^b^	227 (191)	230 (248)	213 (155)	358 (224)
Informal care	6196 (7875)	4008 (6046)	4073 (6107)	6507 (8521)
Lost employment	14,865 (13,115)	13,958 (12,044)	14,638 (13,406)	14,157 (12,568)
*Total health costs*	*2256 (1220)*	*2322 (870)*	*2224 (1073)*	*1424 (1276)*
*Total societal costs*	*23,317 (17,284)*	*20,288 (14,363)*	*20,935 (15,531)*	*22,088 (17,438)*

a see Appendix S1 for servpone.0040808.g003.tifices included in these categories;

b therapy and SMC costs are for 640 participants.

APT  =  adaptive pacing therapy, CBT  =  cognitive behaviour therapy, GET  =  graded exercise therapy, SMC  =  specialist medical care alone.

Informal care costs of patients allocated to APT were significantly higher than for CBT (difference £1580, 95% CI £139 to £3132) and GET (difference £1588, 95% CI £442 to £2694). Patients allocated to SMC also had higher informal care costs than CBT (difference £1165, 95% CI £289 to £2194) and GET (difference £1173, 95% CI £740 to £1569). Lost production costs were significantly higher for APT compared to CBT (difference £1279, 95% CI £141 to £2772). Societal costs (i.e. healthcare, informal care and lost production costs) were significantly lower for patients allocated to CBT compared to APT (difference £2607, 95% CI £432 to £5585). Other differences were not statistically significant.

### Welfare Benefits and Other Financial Payments

Receipt of benefits due to illness or disability increased slightly from baseline to follow-up (Table 4). Patients in the SMC group had the lowest level of receipt at baseline but the figures at follow-up were similar between groups. Relatively few patients were in receipt of income-related benefits or payments from income protection schemes and differences between groups were not substantial.

**Table 4:** N (%) receiving welfare benefits or other financial payments.

Time point	APT(n = 141)	CBT(n = 138)	GET(n = 134)	SMC(n = 143)
*Income benefits*				
6-month pre-randomisation period	28 (18)	16 (10)	22 (14)	17 (11)
12-month post-randomisation period	33 (22)	19 (13)	29 (20)	20 (14)
*Illness/disability benefits*				
6-month pre-randomisation period	42 (26)	51 (32)	50 (31)	34 (21)
12-month post-randomisation period	57 (38)	56 (38)	52 (36)	58 (39)
*Payments from income protection schemes or private pensions*				
6-month pre-randomisation period	10 (6)	9 (6)	13 (8)	8 (5)
12-month post-randomisation period	12 (8)	17 (12)	22 (16)	11 (7)

APT  =  adaptive pacing therapy, CBT  =  cognitive behaviour therapy, GET  =  graded exercise therapy, SMC  =  specialist medical care alone.

### Outcomes

APT, CBT and GET each resulted in improvements in health-related quality of life (measured with the EQ-5D) while SMC produced little change (Table 5). CBT produced the largest QALY gain, significantly more than SMC. After controlling for baseline utility, the difference between CBT and SMC was 0.05 (95% CI 0.01 to 0.09). No other differences between treatment groups were statistically significant. The number (%) of patients achieving a clinically significant reduction in fatigue in each group was: APT 96 (64.0), CBT 113 (76.4), GET 123 (79.9), and SMC 98 (64.9). This difference was statistically significant (Fisher’s Exact test, p = 0.002). The number (%) of patients achieving a clinically significant reduction in disability in each group was: APT 75 (49.0), CBT 105 (71.0), GET 108 (70.1), and SMC 88 (57.9). This difference was also statistically significant (Fisher’s Exact test, p<0.001).

**Table 5:** EQ-5D utilities and QALYs accrued during follow-up period.

Time point	APT(n = 148)	CBT(n = 143)	GET(n = 143)	SMC(n = 151)
Baseline	0.48 (0.27)	0.54 (0.24)	0.52 (0.26)	0.50 (0.28)
12-week	0.53 (0.28)	0.59 (0.25)	0.54 (0.28)	0.52 (0.28)
24-week	0.54 (0.27)	0.61 (0.26)	0.60 (0.29)	0.52 (0.29)
52-week	0.54 (0.29)	0.63 (0.28)	0.59 (0.30)	0.53 (0.31)
QALYs accrued	0.53 (0.22)	0.60 (0.21)	0.57 (0.23)	0.52 (0.25)

APT  =  adaptive pacing therapy, CBT  =  cognitive behaviour therapy, GET  =  graded exercise therapy, SMC  =  specialist medical care alone.

### Cost-effectiveness from a Healthcare Perspective

**Figure 1:**
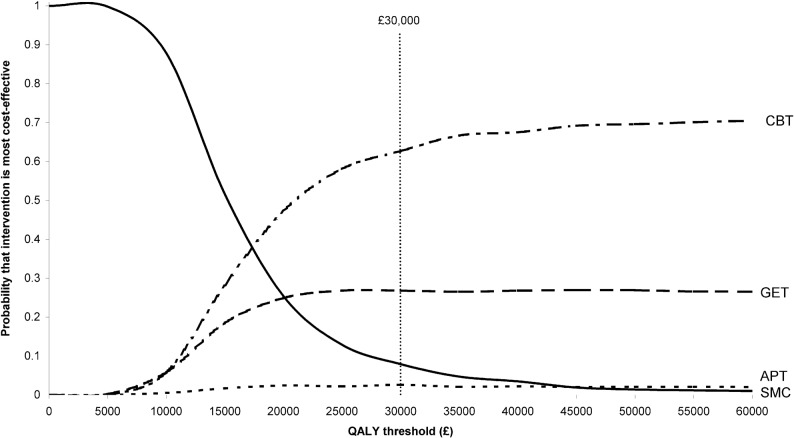
QALY-based cost-effectiveness acceptability curves (healthcare perspective).

At a threshold of £30,000 per QALY, CBT had a 62.7% likelihood of being the most cost-effective option from a healthcare perspective followed by GET at 26.8% (Figure 1). APT had a 2.6% likelihood of being most cost-effective, which was less than the figure for SMC (7.9%).

The ICERs showing the healthcare cost per QALY for CBT and GET compared to SMC were both below the threshold of £30,000 while the ICER for APT compared to SMC was substantially higher (Table 6). The healthcare costs per extra person with a clinically significant reduction in fatigue and disability are also shown. It is clear that achieving such a reduction for one person is associated with a much lower cost, compared to SMC, for CBT or GET than it is for APT. In fact, SMC *dominates* (i.e. has better outcomes and lower costs) APT with regard to disability.

**Table 6:** Cost-effectiveness results from healthcare and societal perspectives, 0–52 weeks.

	CBT v SMC	APT v SMC	GET v SMC
*Outcome: QALYs (n = 570)*			
Incremental effect	0.0492	0.0149	0.0343
Incremental healthcare cost	£904	£823	£810
ICER (healthcare)	£18,374 per QALY	£55,235 per QALY	£23,615 per QALY
Incremental societal cost	−£698	£1893	−£472
ICER (societal)	CBT dominant	£127,047 per QALY	GET dominant
*Outcome: Fatigue (n = 573)*			
Incremental effect^a^	11.1	1.9	14.0
Incremental healthcare cost	£898	£863	£837
ICER (healthcare)	£8105 per person improved	£44,715 per person improved	£5987 per person improved
Incremental societal cost	−£796	£2180	−£400
ICER (societal)	CBT dominant	£112,953 per person improved	GET dominant
*Outcome: Disability (n = 577)*			
Incremental effect^a^	13.4	−8.5	12.6
Incremental healthcare cost	£904	£850	£842
ICER (healthcare)	£6366 per person improved	SMC dominant	£6683 per person improved
Incremental societal cost	−£794	£1948	−£397
ICER (societal)	CBT dominant	SMC dominant	GET dominant

a Percentage point difference between groups.

QALY  =  quality-adjusted life year, ICER  =  incremental cost-effectiveness ratio,

APT  =  adaptive pacing therapy, CBT  =  cognitive behaviour therapy, GET  =  graded exercise therapy, SMC  =  specialist medical care alone.

### Cost-effectiveness from a Societal Perspective

**Figure 2:**
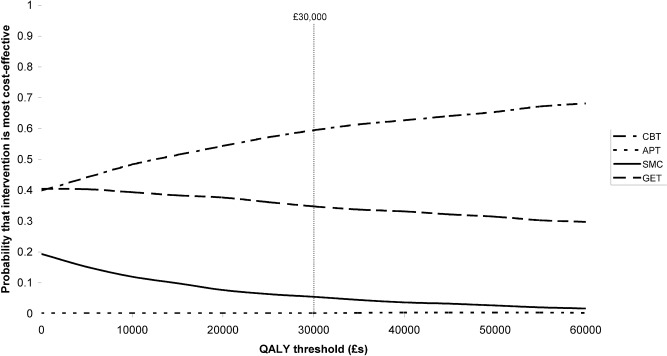
QALY-based cost-effectiveness acceptability curves (societal perspective).

Again at a threshold of £30,000 per QALY, CBT had a 59.5% likelihood of being the most cost-effective option from a societal perspective (Figure 2). GET had a likelihood of 34.8% while APT and SMC had likelihoods of 0.2% and 5.5% respectively.

CBT and GET both dominated SMC from a societal perspective with regard to QALYs gained and reductions in fatigue and disability. Compared to SMC, APT had an incremental cost per QALY substantially higher than the £30,000 threshold while the cost per person with a clinically significant reduction in fatigue was high. SMC dominated APT with regard to disability.

### Sensitivity Analyses

The healthcare costs per QALY gained for CBT and GET compared to SMC were below the cost-effectiveness threshold of £30,000. The cost of CBT would need to increase by 45% and GET by 22% for the cost per QALY to reach £30,000. Therapy costs for APT would need to fall 35% for APT to have a cost per QALY compared to SMC of £30,000. No other sensitivity analyses (i.e. changing the value of informal care, lost employment and standardised medical care) had a large impact on cost-effectiveness.

## Discussion

### Main Findings

We found that adding APT, CBT or GET to SMC resulted in significantly increased healthcare costs. For CBT and GET the healthcare costs per QALY gained were lower than the conventional £30,000 threshold used in England, indicating cost-effectiveness; for APT the cost per QALY was in excess of this threshold.

The major contributors to societal costs were lost employment and informal care costs. The cost-effectiveness of CBT was more apparent from this perspective than from the narrower healthcare perspective; CBT dominated APT, GET and SMC, while GET dominated APT and SMC. Informal care costs were substantial for each group and significantly lower after receiving CBT and GET when compared to APT and SMC. CFS affects patients in many ways and can have a major effect on family members. This study suggests that CBT and GET could ameliorate this effect. However, with the exception of a difference between CBT and APT, there were no significant differences in either lost work time or benefits between the treatments during follow up. In fact, benefits increased across all four treatments.

There are few previous studies with which to compare our results. The additional healthcare costs per QALY gained with CBT and GET compared to SMC alone in this study were £18,374 and £23,615 respectively. These costs are substantially lower than the figure of an additional €51,642 per QALY reported for CBT compared to usual care in a Dutch study [19]. The QALY gain for CBT over usual care in that study was slightly less than we found which may account for some of this difference, given that average costs were similar. Two other studies have assessed cost-effectiveness of treatments for CFS [20], [21]. However, only a minority of patients in both of these studies had CFS and neither used QALYs as an outcome measure.

This study has found that CBT and GET are cost-effective options for treating patients with CFS. However, for patients to benefit from these therapies there needs to be investment to provide the staff trained to deliver them. The findings we report suggest that such investment would be justified in terms of improved quality of life of patients and would actually be cost saving if all costs including societal costs are considered.

### Limitations

The study has limitations. First, we relied on self-reported information on service use and lost employment. There may be issues of accuracy with this approach but it was largely unavoidable given the need for a comprehensive perspective. Other studies have shown this to be an acceptable method [22]–[24]. The accuracy of our cost estimates was enhanced by the direct recording of the number and duration of therapy sessions which formed around half of the costs. Second, average medication costs were assumed for the cost periods. While greater variation would be expected, medication costs are small compared to other service costs. Third, we used the EQ-5D to generate QALY values. This is a recommended method in England, but the sensitivity of the measure in relation to changes in clinical measures in the CFS area has not yet been established. Fourth, we made assumptions regarding the value of unpaid care from family and friends and lost employment. However, sensitivity analyses revealed that the results were robust for alternative assumptions. Fifth, lost employment was valued using the human capital approach. It has been argued that this may overestimate such costs when there is high unemployment and that costs should be confined to the ‘friction period’ during which new employees can be recruited [25]. However, what is evident is that lost work days did not show much difference between the groups and alternative approaches, while possibly reducing absolute societal costs, would not change the cost-effectiveness results. Sixth, there is similar uncertainty around the most appropriate way of valuing informal care [26]. Alternative approaches were used in the sensitivity analyses and these did not make a substantial difference to the results. We adopted the opportunity cost approach and used average wages to reflect this value. Seventh, we analysed data only for those participants where we had data at both baseline and follow-up. This may have introduced some distortions to the results but there were few differences between patients with missing data and those on whom we had complete data. Finally, although one-year follow-up is longer than that obtained in most previous trial of treatments for CFS, we cannot be certain about the longer-term cost-effectiveness of these treatments.

### Conclusions

At a conventional cost-per-QALY threshold considering a one year outcome CBT has the highest probability of being the most cost-effective treatment option for CFS when given as a supplement to SMC and compared to SMC alone. GET has a lower probability of being the most cost-effective option but is more likely to be so than APT or SMC alone. The probabilities that CBT GET are the most cost-effective options at a societal level are higher still, largely due to cost-savings from reducing the care required from family members.

## Supporting Information

Appendix S1 **Unit costs used in PACE study.**
(DOC)
