# Expression of Concern: Adaptive Pacing, Cognitive Behaviour Therapy, Graded Exercise, and Specialist Medical Care for Chronic Fatigue Syndrome: A Cost-Effectiveness Analysis

**DOI:** 10.1371/journal.pone.0177037

**Published:** 2017-05-02

**Authors:** The PLOS ONE Editors

Several readers have raised concerns about some of the analyses reported in the article and made requests for the data underlying this study.

The *PLOS ONE* policy governing the sharing of data that applies to articles submitted before March 3, 2014, requires that authors agree to make freely available any materials and data described in their publication that may be reasonably requested for the purpose of academic, non-commercial research (http://journals.plos.org/plosone/s/file?id=c4aa/PLOSONE_data_policy_before_2014March.pdf).

We assessed the concerns raised and the requests for data and we sought advice from two editorial board members. The advice we received was that the individual-level patient data for Tables 1,2,3,4 and 5 are necessary to replicate the cost-effectiveness analyses reported in the article. In line with the advice received, we contacted the authors to request the individual-level patient data for these tables. The authors raised concerns related to patient confidentiality and specifications under the consent sought from participants at the time of recruitment for the trial.

In consideration of the requirements for ethical oversight of data access that may apply to datasets involving human subjects, we contacted the authors and Queen Mary University of London, where the dataset is held, to request that steps be taken to develop a mechanism that would allow requests for data to be independently reviewed and the data released in accordance with our policy while respecting patient privacy. The authors and Queen Mary University of London shared the data policy in place at the institution, however we consider that aspects of the existing framework impose limitations and conditions not aligned with our editorial policy.

The authors have offered to release aggregated data from the study but have reiterated reservations about the public release of individual-level patient data. The journal policy does not require public release of anonymised patient-level data, but does require a suitable framework for data access for the purpose of academic, non-commercial research. While the release of summarized data does not fully comply with the journal requirements, we welcome that the authors are now willing to share summarized data, and we will provide this once it is made available to us.

In spite of requests to the authors and Queen Mary University of London, we have not yet received confirmation that an institutional process compatible with the existing PLOS data policy at the time has been developed or implemented for the independent evaluation of requests for data from this study. We conclude that the lack of resolution towards release of the dataset is not in line with the journal’s editorial policy and we are thus issuing this Expression of Concern to alert readers about the concerns raised about this article.

## Statement from the authors

We disagree with the Expression of Concern about our health economic paper that *PLOS ONE* has issued and do not accept that it is justified. We believe that data should be made available and have shared data from the PACE trial with other researchers previously, in line with our data sharing policy. This is consistent with the data sharing policies of Queen Mary University of London, and the Medical Research Council, which funded the trial. The policy allows for the sharing of data with other researchers, so long as safeguards are agreed regarding confidentiality of the data and consent as specified by the Research Ethics Committee (REC). We have also pointed out to *PLOS ONE* that our policy includes an independent appeal process, if a request is declined, so this policy is consistent with the journal’s policy when the paper was published.

During negotiations with the journal over these matters, we have sought further guidance from the PACE trial REC. They have advised that public release, even of anonymised data, is not appropriate. As a consequence, we are unable to publish the individual patient data requested by the journal. However, we have offered to provide key summarised data, sufficient to provide an independent re-analysis of our main findings, so long as it is consistent with the REC decision, on the *PLOS ONE* website. As such we are surprised by and question the decision by the journal to issue this Expression of Concern.
