# Supplementary material for: Adaptive Pacing, Cognitive Behaviour Therapy, Graded Exercise, and Specialist Medical Care for Chronic Fatigue Syndrome: A Cost-Effectiveness Analysis
Source: PLoS One. 2012 Aug 1;7(8):e40808. doi: 10.1371/journal.pone.0040808 (PMC3411573; doi:10.1371/journal.pone.0040808)
Supplement: Appendix S1 — Unit costs used in PACE study. (DOC) [file pone.0040808.s001.doc]

**Appendix 1. Unit costs used in PACE study.**

| **Service** | **Cost (2009/10 £s)** | **Unit** | **Source** | |
| --- | --- | --- | --- | --- |
| Interventions |  |  |  | |
| APT | 100 | hour | Calculated for study | |
| CBT | 110 | hour | Calculated for study | |
| GET | 100 | hour | Calculated for study | |
| SMC (based on medical consultant) | 169 | hour | 11 | |
| Primary care |  |  |  | |
| General practitioner | 166 | hour | 11 | |
| Practice nurse | 72 | hour | 11 | |
| Other doctor |  |  |  | |
| Neurologist | 280 | hour | 13 | |
| Psychiatrist | 272 | hour | 11 | |
| Other specialist | 304 | hour | 11 | |
| Health professional |  |  |  | |
| Dentist | 50 | attendance | estimate | |
| Optician | 50 | attendance | estimate | |
| Pharmacist | 100 | hour | 11 | |
| Psychologist | 81 | hour | 11 | |
| Physiotherapist | 42 | hour | 11 | |
| Community mental health nurse | 56 | hour | 11 | |
| Occupational therapist | 42 | hour | 11 | |
| Inpatient |  |  |  | |
| Inpatient (1 day) | 535 | day | 13 | |
| Inpatient (> 1 day) | 404 | day | 13 | |
| Inpatient (<1 day) | 268 | partial-day | 13 | |
| Accident and emergency |  |  |  | |
| Accident and emergency | 97 | attendance | 11 | |
| Medication |  |  |  | |
| Hypnotics | 4.25 | 6-month supply | | 14 |
| Anxiolytics | 4.01 | 6-month supply | | 14 |
| Tricyclic antidepressants | 3.43 | 6-month supply | | 14 |
| Selective serotonin reuptake inhibitors | 3.36 | 6-month supply | | 14 |
| Other antidepressants | 19.13 | 6-month supply | | 14 |
| Analgesics | 7.81 | 6-month supply | | 14 |
| Complementary healthcare |  |  |  | |
| Complementary healthcare | 40 | contact | estimate | |
| Informal care |  |  |  | |
| informal care (based on mean wage) | 14.60 | hour | 15 | |
| Other services |  |  |  | |
| Social worker | 213 | hour | 11 | |
| Support worker | 18 | hours | 11 | |
| MRI | 174 | investigation | 13 | |
| CT | 101 | investigation | 13 | |
| Ultrasound | 55 | investigation | 13 | |
| X-ray | 55 | investigation | estimate | |
| EEG | 115 | investigation | 13 | |
| Blood test | 4 | investigation | 13 | |
| Nutritionist/dietician | 34 | hour | 11 | |
| Massage (costed as physiotherapist) | 42 | hour | 11 | |
| Counsellor | 44 | hour | 11 | |
| Psychotherapist | 81 | hour | 11 | |
| Dental hygienist | 72 | hour | 11 | |
| Other nurse/occupational health | 72 | hour | 11 | |
| Midwife | 73 | hour | 11 | |
| Podiatrist/chiropodist | 27 | contact | 11 | |
|  |  |  |  | |
| Lost employment |  |  |  | |
| Male, age <18, full-time | 36 | day | 15 | |
| Male, age 18-21, full-time | 58 | day | 15 | |
| Male, age 22-29, full-time | 93 | day | 15 | |
| Male, age 30-39, full-time | 141 | day | 15 | |
| Male, age 40-49, full-time | 158 | day | 15 | |
| Male, age 50-59, full-time | 150 | day | 15 | |
| Male, age 60+, full-time | 122 | day | 15 | |
| Male, age <18, part-time | 22 | day | 15 | |
| Male, age 18-21, part -time | 43 | day | 15 | |
| Male, age 22-29, part -time | 78 | day | 15 | |
| Male, age 30-39, part -time | 116 | day | 15 | |
| Male, age 40-49, part -time | 132 | day | 15 | |
| Male, age 50-59, part -time | 131 | day | 15 | |
| Male, age 60+, part -time | 94 | day | 15 | |
| Male, age <18, full-time/part-time | 27 | day | 15 | |
| Male, age 18-21, full-time/part-time | 53 | day | 15 | |
| Male, age 22-29, full-time/part-time | 92 | day | 15 | |
| Male, age 30-39, full-time/part-time | 140 | day | 15 | |
| Male, age 40-49, full-time/part-time | 157 | day | 15 | |
| Male, age 50-59, full-time/part-time | 149 | day | 15 | |
| Male, age 60+, full-time/part-time | 118 | day | 15 | |
| Female, age<18, full-time | 32 | day | 15 | |
| Female, age 18-21, full-time | 51 | day | 15 | |
| Female, age 22-29, full-time | 84 | day | 15 | |
| Female, age 30-39, full-time | 111 | day | 15 | |
| Female, age 40-49, full-time | 110 | day | 15 | |
| Female, age 50-59, full-time | 105 | day | 15 | |
| Female, age 60+, full-time | 93 | day | 15 | |
| Female, age <18, part-time | 21 | day | 15 | |
| Female, age 18-21, part -time | 40 | day | 15 | |
| Female, age 22-29, part -time | 72 | day | 15 | |
| Female, age 30-39, part -time | 93 | day | 15 | |
| Female, age 40-49, part -time | 89 | day | 15 | |
| Female, age 50-59, part -time | 85 | day | 15 | |
| Female, age 60+, part -time | 68 | day | 15 | |
| Female, age <18, full-time/part-time | 23 | day | 15 | |
| Female, age 18-21, full-time/part-time | 46 | day | 15 | |
| Female, age 22-29, full-time/part-time | 82 | day | 15 | |
| Female, age 30-39, full-time/part-time | 107 | day | 15 | |
| Female, age 40-49, full-time/part-time | 104 | day | 15 | |
| Female, age 50-59, full-time/part-time | 99 | day | 15 | |
| Female, age 60+, full-time/part-time | 81 | day | 15 | |
| Male, age <18, full-time | 4.49 | hour | 15 | |
| Male, age 18-21, full-time | 7.90 | hour | 15 | |
| Male, age 22-29, full-time | 11.72 | hour | 15 | |
| Male, age 30-39, full-time | 16.60 | hour | 15 | |
| Male, age 40-49, full-time | 18.50 | hour | 15 | |
| Male, age 50-59, full-time | 17.73 | hour | 15 | |
| Male, age 60+, full-time | 14.89 | hour | 15 | |
| Male, age <18, part-time | 5.64 | hour | 15 | |
| Male, age 18-21, part -time | 6.77 | hour | 15 | |
| Male, age 22-29, part -time | 9.12 | hour | 15 | |
| Male, age 30-39, part -time | 13.43 | hour | 15 | |
| Male, age 40-49, part -time | 15.94 | hour | 15 | |
| Male, age 50-59, part -time | 15.78 | hour | 15 | |
| Male, age 60+, part -time | 12.97 | hour | 15 | |
| Male, age <18, full-time/part-time | 5.08 | hour | 15 | |
| Male, age 18-21, full-time/part-time | 7.60 | hour | 15 | |
| Male, age 22-29, full-time/part-time | 11.54 | hour | 15 | |
| Male, age 30-39, full-time/part-time | 16.48 | hour | 15 | |
| Male, age 40-49, full-time/part-time | 18.41 | hour | 15 | |
| Male, age 50-59, full-time/part-time | 17.64 | hour | 15 | |
| Male, age 60+, full-time/part-time | 14.63 | hour | 15 | |
| Female, age<18, full-time | 4.68 | hour | 15 | |
| Female, age 18-21, full-time | 7.29 | hour | 15 | |
| Female, age 22-29, full-time | 11.56 | hour | 15 | |
| Female, age 30-39, full-time | 15.13 | hour | 15 | |
| Female, age 40-49, full-time | 14.84 | hour | 15 | |
| Female, age 50-59, full-time | 14.35 | hour | 15 | |
| Female, age 60+, full-time | 12.67 | hour | 15 | |
| Female, age <18, part-time | 5.37 | hour | 15 | |
| Female, age 18-21, part -time | 6.63 | hour | 15 | |
| Female, age 22-29, part -time | 8.92 | hour | 15 | |
| Female, age 30-39, part -time | 11.75 | hour | 15 | |
| Female, age 40-49, part -time | 11.34 | hour | 15 | |
| Female, age 50-59, part -time | 10.88 | hour | 15 | |
| Female, age 60+, part -time | 10.26 | hour | 15 | |
| Female, age <18, full-time/part-time | 5.16 | hour | 15 | |
| Female, age 18-21, full-time/part-time | 7.04 | hour | 15 | |
| Female, age 22-29, full-time/part-time | 11.15 | hour | 15 | |
| Female, age 30-39, full-time/part-time | 14.30 | hour | 15 | |
| Female, age 40-49, full-time/part-time | 13.84 | hour | 15 | |
| Female, age 50-59, full-time/part-time | 13.40 | hour | 15 | |
| Female, age 60+, full-time/part-time | 11.54 | hour | 15 | |
